# Emerging Roles for Non-Coding RNAs in Male Reproductive Development in Flowering Plants

**DOI:** 10.3390/biom2040608

**Published:** 2012-12-04

**Authors:** Robert Grant-Downton, Josefina Rodriguez-Enriquez

**Affiliations:** 1 Department of Plant Sciences, University of Oxford, South Parks Road, Oxford OX1 3RB, England; 2 Instituto de Bioorgánica Antonio González (IUBO) University of La Laguna; Avenida Astrofísico Francisco Sánchez; 38206 La Laguna Tenerife, Spain (J.R.-E.)

**Keywords:** reproduction, flowering plant, pollen, gametophyte, fertility, small RNA, non-coding RNA, cytoplasmic male sterility

## Abstract

Knowledge of sexual reproduction systems in flowering plants is essential to humankind, with crop fertility vitally important for food security. Here, we review rapidly emerging new evidence for the key importance of non-coding RNAs in male reproductive development in flowering plants. From the commitment of somatic cells to initiating reproductive development through to meiosis and the development of pollen—containing the male gametes (sperm cells)—in the anther, there is now overwhelming data for a diversity of non-coding RNAs and emerging evidence for crucial roles for them in regulating cellular events at these developmental stages. A particularly exciting development has been the association of one example of cytoplasmic male sterility, which has become an unparalleled breeding tool for producing new crop hybrids, with a non-coding RNA locus.

## 1. Introduction

Male reproductive development in flowering plants is a unique and complex series of events that culminates in the production of highly specialized male gametes (sperm cells). Unlike animals, flowering plants do not segregate a germline early in development and instead differentiate their male reproductive structures late in post-embryonic development directly from established somatic cell lineages [1,2]. Flowering plants are also distinctly different from animals in that the products of meiosis do not directly differentiate into the gametes [2]. Instead of directly forming gametes from the four meiotic products, in male reproductive cell lineages, each of these haploid cells undergoes two further mitotic divisions. This post-meiotic stage of development is called the male gametophyte and is most frequently termed “pollen” [3,4]. Although physically separating from the surrounding somatic anther tissues at an early stage during its development, the male gametophyte is reliant on these cells for protection, support and nutrition, as well as an outer coating of polymeric and other materials. During the later stages of maturation, pollen dehydrates and is liberated from the anther tissues and dispersed into the environment. In this dehydrated form, male gametes can be maintained as viable cells for a considerable period and have the prospect of encountering genetically different female gametes for successful sexual reproduction.

The two mitotic divisions during male gametophyte development form an accessory cell and two sperm cells [3,4]. The initial product of meiosis is called the microspore and this cell undergoes a first highly asymmetric mitotic division (pollen mitosis I). Only one of the cells from this division, the smaller generative cell, will further divide and differentiate into sperm cells. The larger cell that has been generated by pollen mitosis I ceases further cell divisions and becomes an accessory cell. This accessory cell—named the vegetative cell—becomes terminally differentiated and its role at this stage of development is to support the division and differentiation of the generative cell and hence the formation of the sperm cells. The generative cell becomes suspended inside the vegetative cell cytoplasm and here undergoes pollen mitosis II. 

After the dispersed dehydrated pollen encounters a suitable receptive stigmatic surface, the pollen undergoes regulated rehydration and initiates germination. The vegetative cell initiates rapid, highly co-ordinated tip growth to form a “pollen tube” and penetrates the stigmatic surface [5]. Using various signals and cues from the tissues of the stigma and style, the pollen tube grows towards the ovary, containing the female gametes in the ovules [6]. This pollen tube acts as a highly specialized “vehicle” to navigate through the maternal tissues and transport the pair of male gametes contained inside to the fertilization events. Depending on the species, the final mitotic division that generates the sperm can either takes place as the pollen matures in the anther, or it can take place during post-germination pollen tube growth [3,4].

Finally, the pollen tube reaches one of the receptive ovules and, as it enters, it bursts and releases its cargo of two sperm cells [7]. Flowering plants differ from animals in that successful sexual reproduction requires a double fertilization event [8]. One sperm cell fertilizes the egg cell (the female gamete), generating a diploid zygote. The other sperm cell of the pair fertilizes an accessory apparatus to the egg cell, the central cell, which contains two haploid nuclei. This fusion generates a triploid cell that undergoes a specialized developmental program to differentiate into another altruistic and terminally differentiated tissue, endosperm, acting largely as a nutritional support for the developing embryo [9].

**Figure 1:**
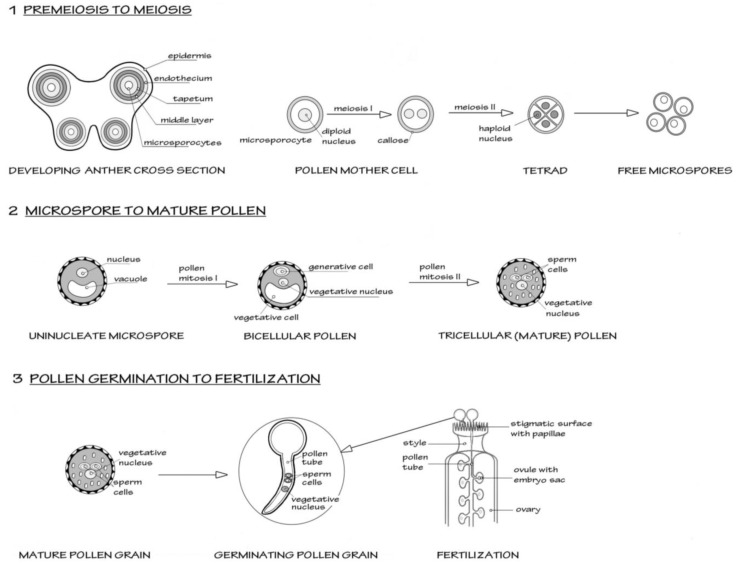
Scheme to show the three major stages in male reproductive development in flowering plants, from premeiosis through to fertilization (syngamy). The model organism *Arabidopsis thaliana* is taken as the basis for this simplified scheme. Section 1 shows the events in the early anther development, where diploid microsporocytes undergo meiosis to form four haploid microspores, the first stage of male gametophyte development. All four microspores survive and further differentiate as shown in Section 2. Two mitotic events occur. The first mitosis (pollen mitosis I) is highly asymmetric and forms a larger vegetative cell and a smaller generative cell. The generative cell then undergoes pollen mitosis II, to form a pair of sperm cells suspended in the vegetative cell cytoplasm. After this mitosis, the pollen undertakes programmed dehydration and is released from the anther. Section 3 shows the events after the pollen grain alights on a suitable receptive stigma. The pollen grain rapidly hydrates and initiates a new phase of growth as the vegetative cell forms a pollen tube. The rapid tip-based growth of the pollen tube penetrates the stigmatic surface and then grows through the tissue of the style towards the ovary. The pollen tubes are attracted to the ovules containing the embryo sac (the female gametophyte). As the pollen tube enters the ovule, it bursts to liberate the two sperm cells. One sperm will form the diploid zygote after fusing with the egg cell. The other sperm initiates triploid endosperm formation after fusing with the nuclei of the central cells.

A simplified schematic of male reproductive development in flowering plants, using the model plant *Arabidopsis*, is shown in Figure 1.

## 2. Evidence for Small Non-Coding RNAs in Male Reproductive Development

As plants do not undergo the transition to the development of sexual reproduction systems until late in post-embryonic growth, there is already a fundamental necessity for small RNA pathways to operate and control somatic development; otherwise, this stage of development cannot be formed or sustained. Hence, certain mutants defective in the general formation and functioning of small non-coding RNAs, such as plants homozygous for strong and null *dicer-like1* (*dcl1*) and *argonaute1* (*ago1*) alleles, have such severe effects on somatic development that normal reproductive development cannot take place, e.g., [10,11,12]. 

At the point when plants make the switch into male reproductive development, little is known about the changes in small RNA systems. Whether plant cells initiating and undergoing meiosis are different with respect to their small RNA systems remains little known. However, one piece of research strongly indicates that this is likely to be the case. In rice (*Oryza sativa*), a specific member of the ARGONAUTE family, *MEIOSIS ARRESTED AT LEPTOTENE1* (*MEL1*), is essential for entry into and progression through meiosis [13]. The exact mode of action of MEL1 remains unknown, for instance it remains undetermined whether the protein binds small RNAs. As *mel1* mutants display altered chromosomal structure and chromatin differences, it is likely that this protein somehow directs developmentally specific chromatin modifications essential for repressing somatic fate and permitting a regular passage through male meiosis. 

Male meiocyte transcriptome studies have shown that these cells are comparatively enriched in known microRNA transcripts [14]. Whether these microRNA precursor transcripts are accompanied by normal processing and formation of mature and functional microRNAs has yet to be demonstrated. The indisputable evidence for small RNAs, for example through sequencing of small RNAs by deep-sequencing methods, has yet to be performed for plant meiocytes let alone isolations of their immediate precursors. This would not only confirm whether corresponding mature microRNAs are abundant, but also would reveal novel meiosis-specific or meiosis-enriched small RNAs.

After meiosis, there is much greater evidence for the operation of small RNA systems in the gametophyte. Following the developmental series from microspore through to mature pollen in *Arabidopsis*, Grant-Downton *et al.* [15] showed that transcripts of many genes involved in small RNA pathways were present in these haploid cells. Interestingly, some transcripts showed unusual dynamics during development such as the RNA-dependent RNA polymerase *RDR5* [15]. The special nature of the small RNA systems of the male gametophyte has been corroborated by other investigations where transcript levels of small RNA pathway genes were analyzed, e.g., [16]. In isolated sperm cells of *Arabidopsis*, transcripts of several small RNA pathway genes were significantly enriched, such as *AGO5*, *AGO9* and *DRB4*, while others were depleted or absent [17]. Recently, investigations of isolated rice sperm cell transcriptomes have also shown that the transcripts of several members of the AGO family are highly up-regulated, along with an RNA-dependent RNA polymerase, *RDR3* [18]. In contrast to *Arabidopsis*, a DICER-LIKE gene involved in biogenesis of 24 nt siRNAs, *DCL3*, is expressed in rice sperm cells [18].

A growing number of studies have shown that small RNAs are present during male reproductive development, largely through high-throughput sequencing-based techniques, such as 454 sequencing and Illumina sequencing. Studies of inflorescence and stamen development suggested that small RNA diversity increases during this phase of development in both dicots and monocots, e.g. [19,20]. Specific studies that have focused on deep sequencing of isolated cell and tissue samples have definitively shown that small non-coding RNAs are present in male reproductive cell lineages. Analysis of mature pollen from *Arabidopsis thaliana* has demonstrated that known microRNAs are unexpectedly diverse [15,21,22,23]. Furthermore, a considerable number of novel microRNAs have been identified from *A. thaliana* mature pollen [21,23]. In pollen development, it is now clear that endogenous microRNA precursor transcripts are transcribed in these cells [15], and that precursor transcripts produced *de novo* in the gametophyte from pollen-specific artificial microRNA constructs are correctly processed into mature microRNAs [16] 

Recently, a developmental perspective on microRNA expression in the male gametophyte has been generated in rice, using deep-sequencing of small RNAs [24,25]. A significant overlap between conserved microRNAs from pollen found in *A. thaliana* and *O. sativa* is seen, suggesting considerable preservation of roles for microRNAs in pollen function between distantly related plant species [24,25]. Given that the developmental process of the male gametophyte is broadly conserved across the angiosperms, this is not surprising. As may be expected, these studies of specific developmental stages in rice have also revealed a considerable number of novel microRNAs. Notably, male gametophyte microRNAs are rather enriched in those known or predicted to target epigenetic pathways such as those affecting chromatin [24,25]. This is fitting with the dramatic and rapid changes that occur in the epigenomic landscape of cells during male reproductive development [2].

Along with conventional microRNAs *A. thaliana* pollen also has been shown to contain a specific class of “phased” siRNAs, called *trans-*acting siRNAs (tasiRNAs) [21] that are initiated by the action of specific microRNAs on long non-coding precursor transcripts [26,27]. These tasiRNAs are then capable of targeting a diversity of coding transcripts for downregulation [26,27]. In pollen, tasiRNAs from *TAS1* and *TAS2* transcripts have been reported, along with their corresponding initiating microRNAs [21]. Interestingly, phased siRNAs initiated by microRNA action on target transcripts (miR2118 and miR2275) have been shown to become highly abundant in male reproductive development in rice, with the greatest abundance in anthers [28]. Interestingly, miR2118 targeting initiates 21 nt phased siRNAs while miR2275 targeting initiates 24 nt phased siRNAs, the former dependent on OsDCL4 and the latter dependent on OsDCL3b [28].

Other classes of siRNAs have also been found in *A. thaliana* pollen. By Illumina sequencing methods, the identification—in both mature pollen and isolated sperm cells—of an unusual abundance of siRNAs derived from transposable element and repetitive DNA sequences has generated considerable interest in the field [16]. This study has shown that pollen vegetative cells derepress transcriptional silencing of TEs and repeats, resulting in transcripts that are processed into short RNAs. Atypically, the derived pollen siRNAs are highly enriched in 21 nt species, rather than 24 nt species normally associated with silenced TEs and repeats [16]. 

## 3. Functional Roles for Small Non-Coding RNAs in Male Reproductive Development

Simply detecting small RNAs in the male gametophyte does not provide evidence that they are functional, as they depend on RNA binding and other interacting proteins to perform functions on target RNA and DNA substrates. MicroRNA-triggered cleavage of target mRNAs has been reported in *A. thaliana* and *O. sativa* pollen for a number of known and new microRNAs [15,21,24,25]. Intriguingly, the microRNA-triggered phased siRNAs reported from rice inflorescence and anther development do not appear to act on their targets through a mechanism of cleavage, and instead may downregulate target transcripts through translational inhibition [28]. The importance of translational inhibition in the male gametophyte itself remains unclear. Although a precise functional role for known or novel microRNAs in post-meiotic development has yet to be identified, the targeting of transcription factors such as those of the AUXIN RESPONSE FACTOR (ARF) family suggests that microRNAs may play a significant role in rapid, dynamic changes to TF transcript levels that occur during development [15,29]. There is a suggestion that miR159 may have a role in *Arabidopsis* pollen development [30] through its action on the MYB transcription factor *DUO1*,with its central role in germline development where it controls a sperm-specific regulon [31,32]. However, detailed analysis of mutants for all three miR159 genes has shown no discernible effect on pollen development or performance [33] and evidence for precise cleavage of *DUO1* in pollen by miR159 is not strong [15].

The study of *A. thaliana* by Slotkin *et al.* [16] has hypothesized that vegetative cell-derived 21 nt siRNAs from TEs and repeats move to the sperm cells nested within vegetative cell cytoplasm, and, in these gametes, act to reinforce transcriptional silencing of corresponding loci. However, the supportive evidence remains tenuous. Even in a species where there is significant physical proximity between a specific sperm cell and the vegetative nucleus, there is no ultrastructural evidence for cytoplasmic continuity between sperm and vegetative cells through serial ultrathin sectioning, only plasmodesmatal connections between the pair of sperm cells [34]. As the pair of sperm cells is surrounded by a second plasma membrane from the vegetative cell, in the absence of proper cell wall structures, the transfer system would have to be specialized. The experimental evidence provided to support this hypothesis [16]—the expression of an artificial microRNA in the vegetative cell targets downregulation of a sperm-cell specific expression of a GFP reporter construct—is not absolutely conclusive proof. The LAT52 promoter used to direct vegetative cell expression of the amiRNA is not restricted to this cell; it is expressed at a lower level in the microspore [35], the precursor to all the cells in the lineage. A further difficulty with this hypothesis is that 21 nt species are implicated in directing RNA-dependent DNA methylation in sperms, whereas evidence from the somatic tissues points to 24 nt species as the major players in guiding this silencing [36]. Another complexity arises from transcriptomic studies of maize (*Zea mays*) sperm cells where it would appear that silencing of TEs had been derepressed as transcripts originating from them made up a notable proportion of their specialized transcriptome [37]. Analysis of maize transposable element data by Vicient [38] showed that transcripts from multiple families of TEs (notably families such as Copia, Gypsy, Cinful, CRM, Prem and Xilon) are highly upregulated in sperm cells compared with other tissues, even other male reproductive tissues. This suggests that full transcriptional silencing of such sequences does not occur in the germline in all plant species. Indeed, a level of epigenetic variation generated in this way during gametogenesis may be important [39], and may in part explain why generation-to-generation differences in the epigenome have been reported recently, e.g., [40]. 

A possible role for one siRNA generated from repetitive DNA that is transcriptionally reactivated in pollen development has been uncovered by recent work [41]. siRNA854, previously identified as a highly conserved microRNA, is derived from the *Athila6* retrotransposon transcript and was shown to downregulate *UPB1b* through targeting the 3’ UTR region. As the UPB1b protein is an RNA-binding protein involved in stress granule formation, it is speculated that siRNA854 may have evolved to inhibit translational repression of retrotransposon transcripts by stress granules [41]. How this might fit with pollen biology and reproductive epigenomics is still unclear; this repressional role may be relictual from when *Athila6* was a more active parasitic retroviral entity [42]. 

Perhaps the most conclusive data for a role for small RNAs in male reproductive development comes from the identification of a natural antisense gene pair that generates small RNAs [43]. The *KOKOPELLI/ARIADNE14* gene pair was identified through an insertion mutant line that disrupted the *KOKOPELLI* gene. This mutant had severe defects restricted to the ultimate stage of reproductive development, double fertilization, with many failed fertilization events. The *KOKOPELLI/ARIADNE14* gene pair is co-transcribed only in the sperm, and this natural antisense gene pair generates small RNAs which significantly downregulate *ARIADNE14* transcript levels. Convincing experimental evidence of *ARIADNE14* cleavage could not be produced, suggesting that the siRNAs may be acting predominantly via translational inhibition. It would appear that lack of siRNA regulation due to disruption of the novel gene *KOKOPELLI* results in high levels of *ARIADNE14* transcript levels in sperm, which in turn disrupts normal fertilization. The exact inhibitory mechanism of higher levels of ARIADNE14 in sperm, a putative E3 ubiquitin ligase family member that lacks enzymatic activity, remains open to speculation. Interestingly, this natural antisense gene pair is very narrowly restricted to the Brassicaceae, indicating that exceptionally rapid evolution of regulatory RNA systems can occur in plant reproductive development. 

## 4. Emerging Evidence for Long Non-Coding RNAs in Male Reproductive Development

Several studies in different plant species have uncovered putative long non-coding RNAs that are upregulated during male reproductive development. For instance, a 828 bp cDNA, *BcMF11*, was cloned from *Brassica campestris* and did not appear to have any coding capacity [44]. This transcript was highly expressed in developing flower buds and anthers; although its expression pattern was not exactly located for specific cell types in these tissues, it was suggested that it may have a role in pollen development. However, another putative long non-coding RNA from maize, *zm401*, was first shown to be expressed in mature pollen [45], and subsequent investigations revealed its expression pattern was specific to male reproductive development, with expression in somatic tapetal cells, as well as in the developing male gametophyte [46]. Transgenic plants downregulating *zm401* transcripts affected normal development of the tapetum and microspore, leading to infertile pollen. This downregulation was shown to affect several key genes required for pollen development. [46] Whether this transcript truly represents a non-coding RNA or whether it codes for a short ORF—as it is predicted to potentially form an 89 amino acid product—will require further investigation.

The reproductive stage may be particularly sensitive to perturbations in long non-coding structural RNAs as work on the *slow walker1* (*swa1*) mutant has demonstratedin *Arabidopsis*. SLOW WALKER1 encodes a WD40 protein that has been implicated in pre-rRNA processing in the nucleolus and normal 18S rRNA biogenesis [47]. *swa1* mutants form normal pollen grains, but the transmission through the male is slightly reduced. As *swa1* mutants also affect female gametophyte development, with disruption of progression through the mitotic divisions that generate the mature female gametophyte, it is possible that subtler effects are seen on mitotic progression in the male gametophyte due to carry-over of SWA1 from the sporophyte or due to other effects such as genetic redundancy in male reproductive development. Mutants in *YAOZHE* (*YAO*), encoding a nucleolar WD repeat protein which is also likely to be involved in 18S rRNA biogenesis, shows some disruption of the first mitotic cell division in female gametophyte development and also has a small effect on male gametophytes [48]. Similarly, another *Arabidopsis* mutant—*gametophyte defective1* (*gaf1*)—that affects processing of tRNA and which also dramatically affects female gametophyte development, has a slight effect on male transmission [49]. GAF1 encodes a predicted sub-unit of the RNAses involved in site-specific processing of pre-tRNAs. While mitotic progression in the female gametophyte is significantly affected, the mutant has only slight negative effects on post-germination performance of the pollen tube. Collectively, this data implicates housekeeping structural RNAs as contributory regulators of male reproductive development.

## 5. Evidence for Functions for Non-Coding RNAs in Male Reproductive Development from Male Sterile Plants

The most exciting and striking recent discoveries in the field have come from dedicated efforts to understand the molecular basis of cytoplasmic male sterility (CMS) in cereals. The incredible importance of cytoplasmic male sterility to plant breeding work comes from the use of lines of male sterile plants in the field, where pollen from adjacent male fertile plants acts as the agent for cross-pollination and mass-production of F1 hybrid seeds without the necessity to undertake the painstaking process of individually emasculating flowers to render them incapable of self-pollination [50]. Two recent studies have uncovered the molecular basis of mutant cytoplasmic male sterile lines in rice and both show the mutation is in a genomic sequence that produces long non-coding RNAs. Zhou *et al.* [51] uncovered the genetic basis of an environmentally dependent CMS locus, *p/tms12-1*, which confers sterility dependent on a shorter photoperiod in one breeding line, and sterility dependent on high temperatures in another breeding line. Fine mapping of this locus revealed the point mutation responsible for this conditional sterility, which did not appear to be in a region generating an ORF. *P/TMS12-1* encodes a long non-coding RNA, which nevertheless generates a 21 nt small RNA (osa-smRNA615912) without having a secondary structure typical of precursor transcripts for microRNAs. This small RNA contains the point mutation in *p/tms12-1*. The mode of biogenesis of the small RNA remains unclear; the authors speculate that the precursor transcript may be converted to a double-stranded intermediate by RNA-dependent RNA polymerase action and then cleaved into small RNAs, instead of conventional microRNA processing. Production of this small RNA peaked in developing inflorescence material at the stage of male reproductive cell lineages entering and undergoing meiosis. However, differences in the expression of the small RNA between wild-type and mutant lines under different temperature and photoperiod regimes were not significant. Bioinformatic analysis revealed a number of predicted target transcripts and these may be responsive to the environment rather than the small RNA itself. Hence, the sterility from the point mutation might result from the reduced efficiency of the small RNA to correctly regulate its target transcript(s) that are differentially expressed under varying environmental conditions. When regulation is not correct, defective reproductive development occurs resulting in reduced male fertility. Soon after the paper of Zhou *et al.* [51], Ding *et al.* [52] reported their work on the same CMS locus, derived from the originator mutant 58S. This confirmed that the mutation was a SNP in a long non-coding RNA that produces small RNAs. However, the interpretation of the mode of action is different as the point mutation altered the RNA secondary structure and reduced transcription of the non-coding transcript in the mutants was recorded specifically under long day conditions. Interestingly, mutant and wild-type plants showed stable differences in promoter methylation at this locus. Further work will be required to completely understand the molecular mechanisms of this unusual and important long non-coding RNA.

The emergence of non-coding RNAs as determinants of plant fertility is, in hindsight, not surprising. Restorer genes for cytoplasmic male fertility include those encoding PPR proteins, a large gene family that includes many known to be regulated by microRNAs and tasiRNAs, e.g., [53,54]. Early work on *Petunia* cytoplasmic male sterility by Frankel [55,56] gave a most unexpected and–long before the age of molecular epigenetics–controversial result, e.g., [57]. Heritable alterations to fertility could be induced by grafting material of *Petunia* with cytoplasmic male sterility onto wild-type plants. Further, the efficiency of transgenerational inheritance of cytoplasmic male sterility in these grafting experiments depended on environmental conditions. Although controversial, it was replicated independently by other workers and thought to be viral in origin [58]. Similar results have been reported for sugar beet [59]. This fascinating work implicates non-coding RNAs–which often have the capacity to move systemically between cells and tissues *in planta* [60] —as well as downstream epigenetic changes induced by non-coding RNA action, such as site-specific heritable alterations to DNA methylation and chromatin [60]. These intriguing early experiments strongly suggest that even the highly regulated, late developmental events of plant reproduction remain substantially influenced by epigenetic signals transmitted via somatic tissues. More remarkable still, these experiments would appear to show that events experienced in one generation can dramatically influence the reproductive fertility of subsequent generations. 

## 6. Outstanding Issues in the Field of Non-Coding RNAs in Reproductive Development

Recent progress in the epigenetics of male reproductive development in flowering plants has left many interesting new questions. Many new small RNAs have emerged from just a few studies of specialized cell types, and it seems likely that further discoveries will emerge in years to come. Undoubtedly, the diversity will expand further, as more cell types are covered by sequencing surveys to even greater depths, and with new approaches, such as improved adapter combinations, to ensure a fuller representation of small RNA populations in the RNA sequencing methods [61]. Studies of CMS in rice have highlighted that classes of non-coding RNAs that do not fit “conventional” classifications are likely to have essential roles in coordinating male reproductive development. As has been uncovered in other organisms, in plants, there is still the possibility that novel or exotic epigenetic systems have yet to be identified in the reproductive cells. Already, there are hints that this is the case. For example, null *dcl1* mutants that should largely abolish microRNA biogenesis are transmitted perfectly through the male with no competitive disadvantage to wild type pollen [12]. This is all the more unexpected as DCL1 is also required for nat-siRNA biogenesis [62] yet the *KOKOPELLI/ARIADNE14* system is not apparently disrupted, although higher levels of *ARIADNE14* transcripts were found in a weak, male fertile *dcl1* mutant allele, *dcl1-9* [42]. Furthermore, no other mutants known to be involved in nat-siRNA biogenesis phenocopy *kok* mutants [42]. Already, Borges *et al.* [23] have suggested that microRNA biogenesis in plant reproduction might utilize proteins other than DCL family members with endonuclease activity, such as AGOs, as has been demonstrated in animals [63]. Perhaps interesting discoveries await detailed analysis of small RNA systems in the male gametophyte, especially using the exceptional mutant and transgenic resources in *Arabidopsis*.

As has been discussed in detail earlier in this review, it is also clear that the matter of activity and silencing of transposable elements and repetitive DNA has to be examined in more detail, especially regarding cell–cell movement of epigenetic information. For instance, there is now evidence that a significant level of upregulation of transposable element transcription occurs on a genome-wide scale in *Arabidopsis* meiocytes [14,64]. Intriguingly, *A. thaliana* meiocytes are also associated with the upregulation of two *ARGONAUTE* family transcripts of as-yet-undetermined functions, *AGO3* and *AGO8* [14]. Interestingly, a sub-set of transposons that are upregulated specifically in meiocytes was correlated with the expression of neighboring genes, an effect which may be generated by local chromatin remodeling [14]. These datasets are also fitting with evidence for transient relaxation of transposon silencing at the initiation of mammalian meiosis and an interesting idea for roles of TE-silencing relaxation in promoting synaptonemal complex formation and local recombination rates [65]. Given the huge importance of maintaining and controlling male fertility in crop production and crop breeding, new work to further increase our understanding of this area of non-coding RNA biology is particularly pressing. 
